# Prolonged complete hematologic response in relapsed/refractory T-large granular lymphocyte leukemia after bendamustine treatment

**Published:** 2016-11-01

**Authors:** R. Rosamilio, V. Giudice, I. Ferrara, S. Annunziata, L. Pezzullo, G. Villani, C. Baldi, R. Guariglia, M. Rocco, C. Selleri

**Affiliations:** 1 Department of Medicine and Surgery, University of Salerno, Baronissi, Italy

**Keywords:** LGL leukemia, bendamustine, chemotherapy, salvage therapy, immunosuppression

## Abstract

T-large granular lymphocyte leukemia (T-LGLL) is a chronic clonal proliferation of effector memory cytotoxic CD3^+^CD57^+^CD56^−^ T cells and the current guidelines suggest immunosuppressive therapy as first-line therapy, but the treatment of refractory/relapsed patients is still challenging due to the lack of prospective studies.

We describe a series of two refractory/relapsed T-LGLL patients successfully treated with bendamustine, a chemotherapeutic agent largely used for B-cell neoplasms, but poorly investigated for the treatment of T-cell diseases. Complete remission (CR) was achieved in 3 and 6 months, respectively, and maintained for at least 20 months. One patient relapsed after a 20-month CR, but she was responsive to bendamustine therapy again, obtaining a further prolonged CR.

Bendamustine as single agent or in combination could be a feasible therapeutic option in refractory/relapsed T-LGLL, especially for elderly patients because of its safety profile.

## I. INTRODUCTION

Large granular lymphocyte leukemias (LGLL) are chronic clonal lymphoproliferations of mature post-thymic TCRαβ^+^ CD3^+^CD4^−^CD5^dim^CD8^+^CD27^−^CD28^−^ CD45RO^−^CD57^+^ T cells (T-LGLL, 85% of cases) or CD3^−^ CD56^+^ natural killer cells (NK-LGLL) [1–5]. T-LGLL usually affects old subjects (> 60 years) with an indolent course and a median overall survival > 10 years [1,5]. At diagnosis, T-LGLL patients show peripheral blood (PB) cytopenias, splenomegaly, bone marrow (BM) infiltration by clonal large granular lymphocytes (LGLs), frequently associated with autoimmune or hematologic disorders (40% of cases) (Table 1) [1,3,5].

Current guidelines of T-LGLL suggest to start treatment only in patients with symptomatic or life-threatening cytopenias (Table 2) [3,4]. Immunosuppressive therapy (IST), with single agent methotrexate (MTX), cyclophosphamide (CTX), or cyclosporin-A (CyA), is the first-line therapy of T-LGLL.

Overall response rate with IST ranges from 40% to 70%, but no standard treatments are clearly defined due to the lack of prospective studies [3–5]. Relapsed patients after IST stop or tapering, as defined in Table 3, are eligible for a second course of immunosuppression with similar or different IST [6]. In T-LGLL patients with IST failure after 4 months, a switch to a second-line treatment is recommended [5,7]. Bendamustine is an alkylating agent that may act as a purine analogue, but the precise mechanism of action is still unclear [8]. This drug is largely used in B-cell non-Hodgkin lymphoma (NHL), but recent studies have focused on refractory/relapsed T cell neoplasm patients, suggesting a role of this drug also in T-cell NHL [9–10].

Here, we report two cases of elderly refractory T-LGLL patients who achieved a prolonged complete remission using bendamustine as salvage therapy.

## II. PATIENTS AND METHODS

In this study, two T-LGLL patients were enrolled after informed consent in accordance with the Declaration of Helsinki [11] and the institutional review boards of the Hematology and Transplant Center, Department of Medicine and Surgery, University of Salerno, Italy. The authors retrospectively reviewed all available medical records.

## III. RESULTS

### Case 1

A 73-year-old woman received diagnosis of T-LGLL in 2003 from other institution. Treatment with low-dose oral MTX, (10 mg/m^2^ weekly) and prednisone (PDN) was started in 2008, due to anemia and moderate thrombocytopenia. After a 4-month therapy, in the absence of a clinical response, oral CTX (100 mg/day) and PDN for 4 months were used as second-line treatment without any response, although this therapy was associated with erythropoiesis-stimulating agents (ESAs). In August 2012, the patient referred to our institution for fatigue, transfusion-dependent severe anemia and neutropenia. The basic clinical characteristics, laboratory data on admission and clinical records of the patient are detailed in Table 4.

In September 2012, oral CyA, (2 mg/Kg/day) and PDN were administered as third-line therapy, but the patient experienced the worsening of cytopenias, the increase in the transfusion support (8–10 packed red blood cell transfusions/month), and positivity of indirect and direct antiglobulin tests. After 8 months of no clinical response, the patient performed a salvage therapy with bendamustine (70 mg/m^2^ for 2 consecutive days every 28 days). *Pneumocystis jiroveci* prophylaxis was carried out with trimethoprim/sulfamethoxazole (TMP/SMX), while ESAs were administered to improve erythropoiesis. After the first bendamustine course, our patient experienced a rapid hemoglobin improvement and transfusion independence, achieving complete remission (CR) within three months. Grade 4 neutropenia was documented after the second course of chemotherapy and treated with granulocyte colony-stimulating factors (G-CSFs). The patient experienced also other complications such as *Escherichia coli*-related urinary tract infection (treated with 500 mg/day oral levofloxacin for 6 days) and hypertransaminasemia associated with vasculitis-like rush of the limbs after the third cycle, which led to the treatment discontinuation and patient re-evaluation. Given that the patient was in CR and showed a minimal BM infiltration (LGLs <10% of total cellularity), bendamustine was definitely discontinued. CR was maintained for 20 months, when the patient showed again anemia; for this reason, a second course of 4 cycles of bendamustine was administered, without any clinical toxicity. At last follow-up, 12 months after the end of therapy, the patient was still in CR.

### Case 2

**Figure 1:**
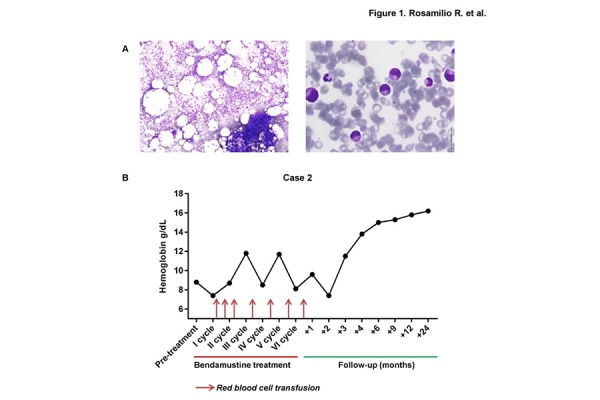
Bone marrow morphological features and clinical course in case 2 T-LGLL. (A) Marrow large granular lymphocytes with abundant cytoplasm and the characteristic azurophilic granules (May–Giemsa assay, Nikon Microscope, Ci-L model; original magnification 20× left panel, and 100× right panel). (B) Hemoglobin levels during and after bendamustine treatment. Red blood cell transfusions are displayed as dark red arrow, according to the time of administration.

A 72-year-old man was admitted to our department in October 2013 for severe anemia associated with atypical circulating and marrow LGLs (Figure 1A). Physical examination did not detected lymphadenopathies and hepatomegaly, but revealed splenomegaly, confirmed by ultrasonography (longitudinal diameter, 150 mm; anteroposterior diameter, 156 mm; transverse diameter, 66 mm). Other clinical characteristics, laboratory data on admission and clinical records of the patient are summarized in Table 4. Immunosuppression was started with CyA (5 mg/Kg/day) and PDN (0.5 mg/kg/day). After a 4-month therapy, the persistence of transfusion-dependent anemia and the worsening of neutropenia (0.29 × 10^9^ cells/L) required a second line therapy. Bendamustine was administrated for a total of 6 cycles at 70 mg/m^2^ for 2 days every 28 days. Bacterial, fungal and *Pneumocystis jiroveci* prophylaxis was carried out using oral levofloxacin (500 mg/day), oral fluconazole (200 mg/day), and TMP/SMX (160–800 mg/twice per week). ESAs and G-CSFs were also administered to reduce myelosuppression and to prevent febrile neutropenia. Grade 3 neutropenia and two episodes of transient hypertransaminasemia with indirect hyperbilirubinemia were documented, but they did not require drug discontinuation. CR was achieved after 6 cycles of bendamustine and, after that, the patient no longer needed transfusions or ESAs administration (Figure 1B). BM aspiration performed at the end of treatment did not show evidence of lymphoproliferative disorder, confirmed by BM flow cytometry immunophenotype and BM biopsy. After 26-month of follow-up, the patient was still in CR.

## IV. DISCUSSION

T-LGLL is a chronic lymphoproliferative disorder with an indolent course, mostly diagnosed after the sixtieth decade of life^1^. T-LGLL patients are a fragile population because of their older age (>60-year-old), the presence of comorbidities (frequently autoimmune disorders) and symptoms which negatively influence the quality of life and the outcome [12,13]. Nowadays, there are no “curative” therapeutic options for T-LGLL. For this reason, the goals are the stabilization of the disease and the correction of cytopenias. IST is the mainstay of treatment for T-LGLL [3,5], but the optimal therapeutic strategy for refractory/relapsed patients is still challenging, even though purine analogues and alemtuzumab are currently used as second-line therapy^4^.

Bendamustine, a molecule developed more than 50 years ago, is an alkylating agent with a complex mechanism of action, sharing characteristics with purine analogues [8,11]. Several mechanisms of action have been hypothesized, as the induction of extensive and durable DNA damage and impairment of DNA repair [8]. The inhibition of the DNA repair mechanism could be enhanced by the *in vitro* combination of bendamustine with cladribine or fludarabine, commonly used for B-cell and T-cell NHL [8,14]. Bendamustine can induce apoptosis through traditional and non-traditional pathways [8]. In particular, the activation of mitotic catastrophe is caspase-2-dependent and cannot be inhibited by overexpression of Bcl-2 [8]. Moreover, the p53-dependent DNA-damage response pathways can be activated not only by bendamustine-induced DNA interstrand crosslinks, but also by oxidative stress and increased release of apoptosis-inducing factor from mitochondria [8]. Based on the interesting results of Zaja et al. in 2013 in a small cohort of T-cell NHL refractory/relapsed patients, we decided to start bendamustine administration in two cases of refractory T-LGLL patients, according to the dosage previously reported [9]. Furthermore, bendamustine was chosen as salvage therapy due to its mechanism of action, hypothesizing that the mitotic catastrophe could avoid the constitutively activated anti-apoptotic pathways in LGLs [8]. Indeed, our series of refractory T-LGLL patients experienced a rapid clinical response and achieved the CR within 3 and 6 months, respectively. One patient relapsed after 20 months of CR with a minimal BM LGL infiltration. Interestingly, she was still responsive to a second course of bendamustine which allowed a 12-months disease-free survival at the time of writing. Moreover, bendamustine was confirmed as a drug with a good safety profile [14], because the myelosuppression was easily managed with G-CSFs and ESAs.

## V. CONCLUSION

Standard salvage treatment for relapsed/refractory T-LGLL patients has not been defined yet due to the lack of prospective studies. Bendamustine-based regimens are increasingly used in both B-cell and T cell neoplasms, but few studies are available for T-LGLL. Our case series shows the efficacy and safety of bendamustine in the treatment of elderly relapsed/refractory T-LGLL patients. Even though these results require further validation in prospective randomized studies, bendamustine as single agent or in combination should be considered a feasible second-line option for relapsed or refractory T-LGLL.

## Figures and Tables

**Table 1:** LGLL diagnostic criteria [3,5]

Clinical presentation: PB cytopenia(s) Splenomegaly Autoimmune diseases LGL > 0.5 × 10^9^ cells/L LGL morphology: Large dimensions (15–18 um of diameter) Round or reniform nucleus Abundant cytoplasm with azurophilic granules LGL phenotype: *T: TCRαβ^+^CD3^+^CD4^−^CD5^dim^CD8^+^CD27^−^CD28^−^CD45RO^−^CD57^+^* *NK: CD3^−^CD16^+^CD56^+^CD57^+^* Presence of clonal TCR rearrangement by PCR or flow cytometry

**Abbreviations.** LGLL = large granular lymphocyte leukemia; PB = peripheral blood; LGL = large granular lymphocytes; TCR = T cell receptor; NK = natural killer cell; PCR = polymerase chain reaction.

**Table 2:** Treatment indications in LGLL [3,5]

Severe neutropenia (absolute neutrophil count <0.5×10^9^ cells/L) Moderate neutropenia (absolute neutrophil count <1×10^9^ cells/L) with recurrent infections Symptomatic or transfusion dependent anemia Severe thrombocytopenia (<0.5 × 10^9^/L) Associated autoimmune conditions requiring therapy

**Abbreviations.** LGLL = large granular lymphocyte leukemia.

**Table 3:** Response criteria in LGLL [3]

**Complete response:** Hemoglobin >12 g/dL Platelet count ≥150 × 10^9^/L ANC >1.5 × 10^9^ cells/L ALC <4 × 10^9^ cells/L with circulating LGLs < 0.5 × 10^9^ cells/L
**Partial response:** Improvements of blood count that do not meet criteria for CR ANC >0.5×10^9^ cells/L or decreasing transfusion requirements
**Treatment failure:** Failure to achieve partial or complete response
**Progressive disease:** Worsening of cytopenias, hepatomegaly or splenomegaly

**Abbreviations.** LGLL = large granular lymphocyte leukemia; ANC = Absolute neutrophil count; ALC = Absolute lymphocyte count; LGLs = large granular lymphocytes; CR = complete remission.

**Table 4:** Patients characteristics

	Case 1	Case 2
• **Age**	73	72
• **Sex**	Female	Male
• **Laboratory data on admission**		
Hemoglobin g/dL	7.7	8.8
White blood cell (× 10^9^ cells/L)	7.01	11.19
Absolute lymphocyte count (× 10^9^ cells/L)	5.41	10.17
Absolute neutrophil count (× 10^9^ cells/L)	1.10	0.615
Platelet count (× 10^9^cells/L)	224	117
Mean corpuscular volume (fL)	112	104
Lactate dehydrogenase (U/L)	644	776
Rheumatoid factor	Negative	n.e.
Anti-nuclear antibodies titer	1:640	n.e.
Direct antiglobulin test	Negative	Negative
• **Peripheral blood LGLs**		
Percentage (%)	75	78
Flow cytometric phenotype	CD3^+^CD8^+^CD7^+^CD5^−^CD4^−^CD56^−^TCRαβ^+^	CD3^+^CD8^+^CD2^+^CD7^+^CD4^−^CD5^−^CD56^−^ TCRαβ^+^
• **Indications for treatment**	Transfusion dependent anemia	Transfusion dependent anemia, splenomegaly
• **Immunosuppressive therapies**	MTX+ PDN, CTX+PDN, CyA+ PDN	CyA+PDN
• **Response to immunosuppressive therapies**	Treatment failure	Treatment failure
• **Time to-BENDA treatment**	10 years	4 months
• **BENDA dosage**	70 mg/m^2^ for 2 days every 28 days	70 mg/m^2^ for 2 days every 28 days
• **Number of BENDA cycles**	3 and 4*	6
• **Response to BENDA**	Complete response	Complete response
• **Follow up (months) after BENDA treatment**	20 and 12∘	26

**Abbreviations.** n.e. = not evaluated; LGLs = large granular lymphocytes; MTX = methotrexate; PDN = prednisone; CTX = cyclophosphamide; CyA = cyclosporine-A; BENDA = bendamustine.

**Symbols**.

* = 3 cycles of BENDA and 4 after relapse;

∘ = 20 months after first BENDA treatment and 12 after the second BENDA treatment.
